# Empyema: Etiology, Symptoms, Treatment and When to Perform Thoracotomy, Report of Cases

**Published:** 1908-10

**Authors:** John T. Burrus

**Affiliations:** High Point, N. C.


### EMPYEMA: ETIOLOGY, SYMPTOMS, TREATMENT AND WHEN TO PERFORM THORACOTOMY, REPORT OF CASES.*

BY JOHN T. BURRUS, M. D., HIGH POINT, N. C.

In looking over the records of papers and reports of cases before the North Carolina Medical Society, I am unable to find a report of Empyema or any article on the subject, and since I have had a number of cases, I have taken this subject for my text. Therefore I invite your attention to this subject for a short while.

The term Empyema, denotes the presence of pus in the pleural cavity, (or pleural abscess). We have two classes of cases, that of Empyema in childhood and in adults. In childhood it is very plain and the symptoms enable us to make a very early diagnosis.

In the adult the diagnosis is very much more difficult to make, and usually requires a longer time than it does in childhood.

*Etiology.*—The causes of Empyema are due to the presence of pyogenic-bacteria in the pleural cavity, and is almost always secondary to some other disease.

1. Secondary to Sero-Fibrinous effusions where Thoracentesis has been performed for Sero-Fibrinous effusion and antiseptic measures have not been strictly adhered to, and secondary to pneumonia, scarlatina, pyemia, tuberculosis, dysentery and some times measles, whooping cough, carious ribs, carious vertebra and trauma.

2. Lymphatic metastasis is a probable way in which bacteria reaches the pleura, from neighboring tissues. The organisms which in most every instance has been found to produce this condition is the micrococcus lanceolatus, streptococcus, staphylococcus and tubercle bacillus.

*Symptoms.*—The history of the patient is important and my experience with the disease shows that 90 per cent of all cases occur after pneumonia or plurisy.

*Read before the North Carolina Medical Society, at Winston Salem.

Where the case runs an ordinary course of pneumonia or pleurisy and perhaps the patient beegins to improve, fever reducing and at the time apparently begins to convalesce. For a few days the patient has every indication of doing well, when a chill comes on followed by fever and pain in side. The symptoms are at first looked upon as a relapse. In a few days dyspnoea, restlessness and immobility of the affected side.

In childhood the symptoms are more rapid in developing than in the adult. In a few weeks the clinical picture is changed. The patient that was once plethoric now becomes very much emaciated. The short loose cough suggesting a tubercular condition, which is in many cases confirmed when the night sweats appear. The general appearance of the patient is that of extreme exhaustion.

Physical signs are: Pains in the affected side, dyspnoea and evidence of absorption of pus, skin cold and clammy and oftimes bathed in perspiration. Respiration is from 36 to 50 to the minute. Temperature varying from 101 F. to 105 F.

There is a dullness in the affected side with change of sound according to the position of the patient. A disappearance of vocal fremitus on the affected side.

The bronchial murmur may be perceptible if the accumupation is not too extensive, but if the effusion is sufficient to occupy most of the pleural space, then bronchial murmur is not heard.

In childhood the bulging of the affected side is not so marked as in the adult, owing to the thoracic viscera being less resistive and more easily displaced as in childhood.

On measuring the side from the center of sternum to the spinus processes, the affected side is seen to be larger, varying according to the amount of pus in the cavity.

In some cases we have the rupture into the bronchi of pus, which is expectorated, and occasionally this relieves the patient, and recovery is the result.

*Treatment.*—In cases of empyema I have very little faith in leaches, blisters, poultices or the other external appliances which have been suggested; neither do I believe in waiting for these cases to rupture or to go so long that the bulging is so extensive that it is ready to rupture externally. But as soon as a diagnosis can be made I think that every case should be operated on.

Usually we allay pain, that our patient may be more comfortable; give a mercurial purge, supporting the heart by giving heart stimulants, and diffusable stimulants such as ammonia, strychnine, antrophine, and digitaline. But the only thing that I have done in these cases, that gave me any results was thoracotomy as early as I could make the diagnosis. And the positive diagnosis is made by the introduction of a trocar obtaining pus.

The positive diagnosis made, I next proceed to do thoracotomy. This should be done by making an incision two and one-half inches long in the six or seven interspace in the mid-axillary line. First observing the strictest antiseptic measures.

If the space is large enough to admit a large size rubber tube, then it is sufficient, but if not, you should resect a rib, which is done in the usual way. This gives a space sufficient to allow free drainage. The tube should be held in situation by ligatures. To the drainage tube there should be a long tube connected, which conveys the fluid from the drain to a vessel containing an antiseptic solution. This acts for two purposes; first, preventing the induction of air, dust or any infection into the pleural cavity, and secondly prevents the dressings from becoming soiled by the drainage, thereby making it more comfortable for the patient.

The after care of the patient demands a consideration. First, stimulate and nourish, second, to see that the tube does not become obstructed, thereby facilitating the free escape of the fluid.

So long as the flow is free, I never irrigate the cavity, but if the flow is very tenacious and does not flow freely, then a weak solution of permanganate of potash, or a normal salt solution is used to irrigate the cavity.

Strych-nitrate syr-hyphophos and nourishment are always instituted and the patient urged to take plenty of fresh air.

Thoracotomies, case No. 1.—Mollie J., age 11 years, white, history good, no scrofula, syphilis or tuberculosis in family. Was taken ill December 15, 1907, with chill. Fever 105 F., 130, high arterial tension, a rapid respiration, and severe pain in left side, complaining of aching all over body. I examined the patient on evening of same date and found her with pneumonia of left lung.

The case run a rather severe course to the 8th day, when fever terminated by crises, and patient began to improve.

On the 14th day I dismissed the case, as the patient was now sitting up and very desirous of food, the cough having almost subsided by this time.

Six days later I was called in and found that 3 days after I dismissed the case she had developed a chill, rapid respiration and a good deal of fever, having a severe cough and not able to expectorate anything. Dyspnoea marked and an inability to lay off of the affected side.

I found upon examination that there was obliteration of inter-costal spaces and heart was displaced to right as far as the median line with dullness over the entire left side.

I introduced an exploring needle into the thoracic cavity and obtained nothing. Whereupon I did not advise an operation.

Four days later I made another exploratory puncture and obtained a small amount of pus. I advised an immediate operation and on the following day Dr. Duncan and myself did a thoracotomy, going through the sixth interspace, between the mid and post axillary line. We introduced a drain and while at first the discharge was very slight, owing to the tenacious pus, but by the third day the patient was discharging large quantities of pus through the tube.

The tube remained in situ for three weeks and was removed. The patient is well and in a fine physical condition, suffering no discomforts from the side, and has made a speedy and uneventful recovery.

Case No. 2.—A. AL. s. child, female, age 7 years. Family history good. Father and no mother living, two brothers and one sister, none dead. Child has never been sick, except measels at the age of two years, from which she had recovered.

January 25, patient was taken ill with pneumonia, which was treated by another physician. On April 1st, Dr. Dorsett was called and diagnosed empyema and urged an imemdiate operation. The family objected to this and on the 14th, I saw the case with Dr. Dorsett and obtained the following history.

On January 25, 1908, child was taken ill with pneumonia in left side and was very sick for two weeks, then she began to improve, and at the end of the third week sat up in bed and called for something to eat.

At this time she had no elevation of temperature and was getting better. At the end of the fourth week patient had a chill and a slight rise of temperature, pain in side and difficult breathing, a dry cough that worried her a great deal, but was unable to expectorate anything.

The case went on in this way till the end of the 8th week. Child spat up quite a quantity of what the parents said looked like pus. Then she improved for a week, but from that time she began to grow worse up to the time I saw her, April 14, saw case and found extreme emaciation, temperature 101 F, pulse 140, respiration 46. Skin cold and clammy and bathed in persipration. A very anxious expression on her face and every few minutes she would have a paryoxism of a very severe cough and was unable to expectorate.

Physical examination revealed the following. Heart displaces two and one-half inches to right of sternum and upwards, obliteration of intercastal spaces on left side and oedematous.

The measurements revealed the left side two inches larger than right. The abdomen was distended and the child was nothing but a bony frame covered integument.

On April 15th, Dr. Dorsett and myself did a thoracotomy, using very rigid antiseptic precautions, making an incision two inches long in the seventh interspace in the mid-axillary line.

A large rubber tube was introduced and anchored by ligatures. We removed five pints of liquid pus. The child's abdomen reduced and the heart became less frequent.

The tube remained in situ three weeks and drained freely all the time. Then we changed the drainage tubes, using a smaller one, and irrigated with a weak solution of hydrogen di-oxide. The tube remained in situ for three weeks longer, making six in all. When there was no further drainage it was removed.

After the operation the child was put on Syr. of Hydriodic acid nutritious diet and the open air.

This case made a good recovery and has gained a great deal of flesh. I report the two cases to show the difference where earlier operation is instituted and where the case has been allowed to remain so long without surgical intervention.

